# Risk Reduction Behaviors Regarding PM_2.5_ Exposure among Outdoor Exercisers in the Nanjing Metropolitan Area, China

**DOI:** 10.3390/ijerph15081728

**Published:** 2018-08-12

**Authors:** Lilin Xiong, Jie Li, Ting Xia, Xinyue Hu, Yan Wang, Maonan Sun, Meng Tang

**Affiliations:** 1 School of Public Health, Southeast University, Nanjing 210003, China; hzxionglilin@163.com (L.X.); yanwang829@foxmail.com (Y.W.); 2 Department of Environmental Health, Nanjing Municipal Center for Disease Control and Prevention, Nanjing 210003, China; 3 School of Civil Engineering, Shenzhen University, Shenzhen 518060, China; jennilion@163.com; 4 Insurance Work and Health Group, Faculty of Medicine Nursing and Health Sciences, Monash University, Melbourne 3003, Australia; ting.xia@monash.edu; 5 Department of Construction Management, Nanjing Forestry University, Nanjing 210037, China; structural_0513@163.com (X.H.); smn1995@foxmail.com (M.S.); 6 Jiangsu Key Laboratory for Biomaterials and Devices, Southeast University, Nanjing 210009, China

**Keywords:** risk reduction behavior, PM_2.5_, outdoor exercisers, influencing factors, China

## Abstract

*Aims*: This study aimed to describe risk reduction behaviors regarding ambient particulate matter with a diameter of 2.5 μm or less (PM_2.5_) among outdoor exercisers and to explore potential factors influencing those behaviors in the urban area of Nanjing, China. *Method*: A cross-sectional convenience sample survey was conducted among 302 outdoor exercisers in May 2015. Descriptive analysis was used to describe demographics, outdoor physical activity patterns, knowledge of PM_2.5_ and risk reduction behaviors. Multivariate logistic regression analysis was then used to explore factors that influence the adoption of risk reduction behaviors. *Results*: The most common behavior to reduce PM_2.5_ exposure was minimizing the times for opening windows on hazy days (75.5%), and the least common one was using air purifiers (19.3%). Two thirds of respondents indicated that they wore face masks when going outside in the haze (59.5%), but only 13.6% of them would wear professional antismog face masks. Participants adopting risk reduction behaviors regarding PM_2.5_ exposure tended to be females, 50–60 year-olds, those with higher levels of knowledge about PM_2.5_ and those who had children. *Conclusions*: These findings indicate the importance of improving knowledge about PM_2.5_ among outdoor exercisers. Educational interventions should also be necessary to guide the public to take appropriate precautionary measures when undertaking outdoor exercise in high PM_2.5_ pollution areas.

## 1. Introduction

The increasing smog occurrences characterized by high fine particulate matter (PM_2.5_) is currently becoming a major health concern in major Chinese cities [1,2,3,4,5]. PM_2.5_ represents the fine particulate matter with aerodynamic diameter of 2.5 μm or less, deriving from vehicular exhaust, fuel burning and some industrial activities [6,7]. Short-term and long-term exposure to PM_2.5_ can cause a variety of adverse health outcomes, such as increased morbidity and mortality relating to both cardiovascular and respiratory diseases [4,8,9,10]. It is therefore important for the public to take actions to reduce PM_2.5_ exposure on days when smog is expected.

In modern society, physical activity has become increasingly prevalent, especially outdoor activities such as walking, running and dancing, in the morning or evening peak hours [11]. Outdoor physical activity is already a culturally rooted daily lifestyle for Chinese people [12]. Regular exercise has been shown to improve overall health and wellbeing, while physical inactivity is the fourth leading risk for global mortality, contributing to 3.2 million deaths annually [13,14,15]. However, outdoor physical activity can amplify respiratory uptake and make deposition of air pollutants faster, deeper into the respiratory system, resulting in the increasing risks of respiratory or cardiovascular diseases with outdoor exercise when air pollution or smog, especially for elderly populations [12,16]. Cardiopulmonary daily mortality is estimated to increase by 6–13% per 10 µg m^−3^ of PM_2.5_ [17,18]. Therefore, it is imperative for those who undertake outdoor exercise to understand individual protective behaviors regarding PM_2.5_.

Previous studies suggest that taking appropriate protective measures can minimize the adverse health effects of PM_2.5_ exposure [9,19,20,21,22], such as wearing professional dust masks, using air filtrations, reducing the duration of opened windows in the fog and haze, regulating daily activities according to the air quality index (AQI), and cleaning the nasal cavity after returning home. However, most studies on risk reduction behaviors regarding PM_2.5_ exposure mainly focus on limited behaviors such as wearing a mask or adjusting outdoor activity time. There is little evidence about multiple/combined risk reduction behaviors relating to PM_2.5_ exposure among outdoor exercisers in China. In particular, utilization of air filtration and cleaning the nasal cavity were rarely reported. Additionally, current findings on some risk reduction behaviors in Chinese population are controversial. For instance, a study conducted by Zhao reported that only 3.9% of urban residents wore face masks during high PM_2.5_ days [23], while another study conducted in Ningbo, a coastal city located on the eastern coast of China, revealed that 48.5% of their respondents wore face masks in the fog and haze. In order to reduce the risk of PM_2.5_ exposure on high pollution days, it is important for healthcare providers and policy-makers to understand how outdoor exercisers currently minimize their exposure to PM_2.5_.

Current literature suggests that a variety of factors influence people’s response to PM_2.5_ exposure and behavior change, such as gender, age, education, household socioeconomic status, and perception of air pollution [24,25,26,27,28]. However, other factors such as home situation and health status are rarely studied. Furthermore, researchers often analyze these risk reduction behaviors and their influencing factors separately. A comprehensive picture of multiple behaviors in evaluating risk reduction behaviors has not been fully described, and neither has the influencing factors of risk reduction behaviors regarding PM_2.5_. Therefore, the overall aims of this cross-sectional case study were to (1) describe major risk reduction behaviors regarding PM_2.5_ exposure among outdoor exercisers in a Chinese setting and (2) explore the potential factors that influence the adoption of risk reduction behaviors by outdoor exercisers.

## 2. Materials and Methods

### 2.1. Study Design

This cross-sectional study was conducted in May 2015 in Nanjing. Nanjing, the capital city of Jiangsu Province, located in eastern China, is one of the most developed and fastest growing urbanized areas. With rapid social and economic development, Nanjing has been facing the challenges of severe air pollution [3,5,10,29,30,31,32,33,34]. There were 242 haze days (i.e., with horizontal visibility less than 10,000 m) in 2013 and increasing red alerts for severely high levels of air pollution were reported in 2015 [35]. There are three possible conditions when the red alerts of haze days are set based on the daily average visibility, relative humidity and PM_2.5_ concentration simultaneously: (1) Haze with visibility less than 1000 m and relative humidity less than 80%. (2) Haze with visibility less than 1000 m and relative humidity no less than 80% with PM_2.5_ concentration ranging 250–500 µg m^−3^. (3) Haze with visibility less than 5000 m and PM_2.5_ concentration over 500 µg m^−3^.

**Figure 1:**
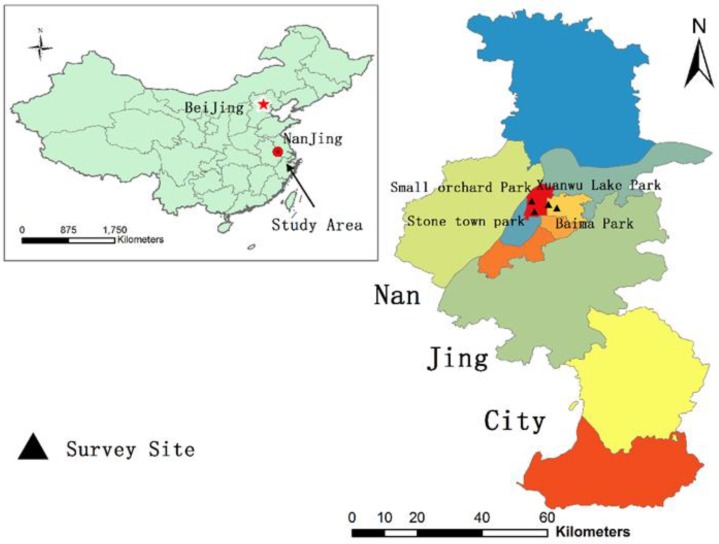
The site distribution of the questionnaire survey.

A survey was developed to explore outdoor exercise participants’ risk reduction behaviors regarding PM_2.5_ exposure and the potential factors that influence the behavior adoption. We conducted the survey during the morning and evening hours, where a high proportion of outdoor exercisers were found in the pilot study. According to data from the local meteorological department, the daily average PM_2.5_ concentration was 51.23 ± 21.63 µg m^−3^ over the survey month in Nanjing, with five days less than 30 µg m^−3^ and only one day peaking at 111 µg m^−3^. Convenience sampling was used to recruit participants at outdoor parks. Two districts, Gulou and Xuanwu, were initially selected, as they are main residential areas representing 23.4% population in the central metropolitan Nanjing [36]. The PM_2.5_ levels were relatively lower than other local districts. Secondly, four parks (i.e., Xuanwu Lake Park, Baima Park, Stone Town Park, and Small Orchard Park) in these two districts were selected to recruit participants, since they were identified as dominant exercise locations based on the pilot study (Figure 1). A total of 302 participants agreed to participate in the survey through face-to-face interview. Interviews were conducted by rigorously trained senior students. All participants were informed of the aim and intent of the study. Written, informed consent was obtained before the surveys were administered. The study was conducted in accordance with the Declaration of Helsinki, and the protocol was approved by the Independent Ethics Committee for Clinical Research of Zhongda Hospital, Affiliated to Southeast University (2014ZDSYLL133.0). 302 questionnaires were successfully collected with 292 participants completing all questions.

### 2.2. Data Collection and Measures

The questionnaire design was guided by the COM-B framework. The COM-B framework is a ‘behavior system’ (B) involving three essential conditions, viz. capability (C), opportunity (O), and motivation (M), developed from the existing theories of behavior change [37,38]. ‘Capability’ is defined as the individual psychological (e.g., knowledge, understanding) and physical capacity to engage in the targeted activity. ‘Opportunity’ refers to all the external factors that make the behavior possible or prompt it, and ‘motivation’ to the mental processes that energize and direct behavior includes beliefs, attitudes and habitual processes and emotional responses [37]. Based on the COM-B framework and the specialties of local outdoor exercisers, four main components were included in the final questionnaire: (1) Socio-demographics (e.g., age group, gender, home situation, health status, and individual income); (2) outdoor physical exercise patterns (e.g., types of physical exercise, and exercise schedule); (3) knowledge about PM_2.5_ (e.g., questions about the connotations of the AQI, the definition of PM_2.5_, the sources and hazards of PM_2.5_, and the components of PM_2.5_); and (4) risk reduction behaviors taken to reduce outdoor air pollution exposure including adjusting physical exercise time/frequency according to the AQI, adjusting physical exercise styles/amounts according to the AQI, the wearing of face masks when going outside in the haze, the cleaning of mouths and noses after outdoor activities, opening windows, and using air purifiers.

After preliminary research and a pilot study, behavioral questions were designed based on the relatively common six risk reduction behaviors regarding PM_2.5_ [9,19,20,21,22,23]. A preliminary investigation with open and closed-ended questions was conducted to identify risk reduction behaviors related to PM_2.5_ exposure and their potential influencing factors. Twenty participants, randomly selected from the study area, took part in the pilot study in early March 2015. Based on the results, questions that were unclear or problematic were revised.

The participants’ knowledge about PM_2.5_ was measured by a 5-point Likert scale, ranging from 1 (very unfamiliar) to 5 (very familiar). The risk reduction behavior items were also measured by 5-point Likert scale, ranging from 1 (never) to 5 (very often). The total scores of items regarding knowledge of PM_2.5_ were then calculated and classified into four levels, from ‘very high’ (scores from 21 to 25), to ‘very low’ (scores from 5 to 10). We created a binary variable using mean value as a cutting point for further analysis. The variable was coded as ‘0’ representing ‘lower-level’ of risk reduction behavior (referring to participants whose total scores of behavior items were below the mean), while the code ‘1’ represented ‘higher-level’ of risk reduction behavior (referring to those whose total scores of behavior items were above the mean). The mean score of 19 was selected as the final cutting point in the final model.

### 2.3. Statistical Analysis

The collected data were double-entered into a database using EpiData 3.1 (EpiData Association, Odense, Denmark). All analyses were performed using SPSS version 18.0 (IBM, Armonk, NY, USA). Descriptive statistics were firstly used to illustrate the demographic characteristics and the percentages of categorical variables. Then, the total scores were calculated for describing respondents’ overall risk reduction behavior regarding PM_2.5_ and the extent of knowledge of PM_2.5_. A multivariate logistic analysis was used to investigate the predictors of participants’ risk reduction behaviors. Predictor variables were socio-demographic characteristics, including age group, gender, occupation; whether or not household with children and self-reported the extent of knowledge about PM_2.5_. Dummy variables were created to represent different attributes of each independent variable, with reference set based on the method of *Indicator* (i.e., the last categorical variable set as reference default in SPSS) during the regression analysis. Significance was defined as *p* < 0.05.

## 3. Results

### 3.1. Socio-Demographic Characteristics of the Study Population

Of the 302 respondents, 52.3% (158) were males. The mean age of the respondents was 60.8 ± 12.2 years-old, with a range from 26 to 86 years old. 22.5% (68) respondents had college diplomas or above. 27.5% (82) of the respondents reported that their individual income per month was less than RMB 3000, whilst 33.5% (100) indicated that they had monthly incomes more than RMB 5000. Around half of the respondents (53%, n = 160) suffered from chronic illnesses, including respiratory diseases (3.3%), cardiovascular and cerebrovascular diseases (41.7%), tumors (0.7%) and other chronic diseases (7.3%). 14.8% (44) of them had children in their household, and 49.3% (148) had a family member with chronic illness (see Table 1).

### 3.2. Outdoor Physical Exercise Habits

Table 2 summarizes the respondents’ outdoor physical exercise patterns. 61.7% (184) of the respondents exercised on a daily basis. Almost 95% (284) of them indicated that they engaged in outdoor exercise for more than 30 min each time, with 43.6% (130) spending 1 to 2 h and 36.9% (110) 0.5 to 1 h. Additionally, nearly 90% (271) of the respondents undertook outdoor physical exercise during the morning (before 8 a.m.) and evening peak hours (after 6 p.m.). Light outdoor exercise such as walking was the most common form of exercise among the respondents (39.1%). Around one third (88) of them participated in two or more types of exercise, such as walking and running or brisk walking and dancing.

### 3.3. Self-Reported Extent of Knowledge about PM_2.5_

**Figure 2:**
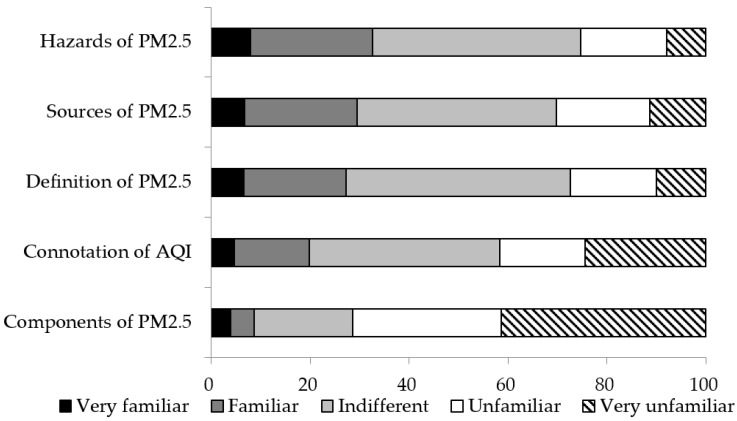
The distribution of self-reported the extent of knowledge about PM_2.5_.

The knowledge of respondents regarding PM_2.5_ is shown in Figure 2. Only 27.3% (82) of respondents reported that they were ‘very familiar’ or ‘familiar’ with the definition of PM_2.5_. Approximately one third (‘sources’, 88, ‘hazards’, 98) of them were ‘very familiar’ or ‘familiar’ with the sources and hazards of PM_2.5_ respectively. Almost one fifth (60) respondents indicated that they were ‘very familiar’ or ‘familiar’ with the connotation of AQI. Only 8.7% (26) were ‘very familiar’ or ‘familiar’ with the components of PM_2.5_.

The average comprehensive score of respondent’s knowledge about PM_2.5_ was 13.57 (SD = 4.2). Overall, 5.5% (16) of the respondents were classified into the group with ‘very high’ knowledge about PM_2.5_, and 22.6% (66) of them were classified into group with ‘very low’ knowledge about PM_2.5_.

### 3.4. Risk Reduction Behavior of PM_2.5_

**Figure 3:**
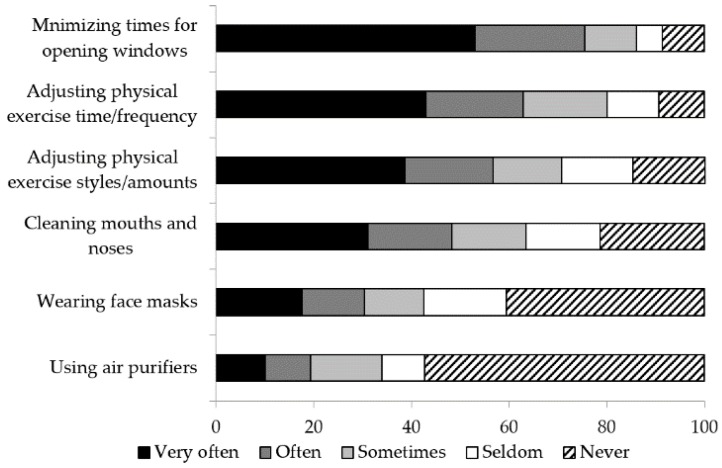
The distribution of risk reduction behavior of the study population.

As shown in Figure 3, around three quarters of respondents (75.5%, n = 228) minimized the times for opening windows, since the majority hoped to directly reduce the PM_2.5_ exposure from outside. More than half of the respondents (62.9%, n = 190) adjusted time of day or frequency that the physical exercise was done “often” or “very often” according to the AQI. 56.7% (170) of respondents adjusted their physical exercise styles or amounts in relation to the AQI “often” or “very often” whilst 14.7% (44) of them ‘never’ did it. Approximately one third of respondents (30.4%, n = 90) wore face masks “very often” or “often” when going outside in the haze. Specifically, among those who wore face masks, nearly half of the respondents (48.9%, n = 86) would wear medical one-off masks, 36.4% (64) would wear cotton or gauze face masks, and only 13.6% (24) would wear professional antismog face masks.

Furthermore, 48.3% (146) of respondents indicated that they would clean their mouths and noses after outdoor activities ‘very often’ or ‘often’ whilst only 19.3% (58) of them would use air purifiers ‘very often’ or ‘often’ when airing the room. Figure 3 shows that the most common way to reduce PM_2.5_ exposure was minimizing times for window opened and the least common one was using air purifiers.

### 3.5. Factors that Influence the Adoption of Risk Reduction Behavior Regarding PM_2.5_

Table 3 shows the results of the multivariate logistic regression analyses relating to factors that influence the risk reduction behavior regarding PM_2.5_. Compared to males, females were more likely to adopt risk reduction behavior against PM_2.5_ risks (OR = 3.22, 95% CI = 1.53–6.78, *p* < 0.05). Respondents between the age of 50 to 60 years old were also more likely adopt risk reduction behavior than those who were younger than 50 years old (OR = 2.53, 95% CI = 1.10–5.85, *p* < 0.05). However, elderly people over 70 years old were also found to be less likely to adopt such behavior, although it is not statistically significant (*p* > 0.05). Compared to respondents with no children in their household, those who had children were 2.56 times more likely to adopt risk reduction behavior (OR = 2.59, 95% CI = 1.07–6.26, *p* < 0.05). In addition, the levels of knowledge about PM_2.5_ was found to be a significant predictor of having a ‘higher-level’ risk reduction behavior in response to PM_2.5_ risks (‘very high’, OR = 12.70, 95% CI = 2.14–75.17, *p* < 0.05. ‘high’, OR = 7.55, 95% CI = 2.64–21.60, *p* < 0.05).

## 4. Discussion

This study described six major risk reduction behaviors regarding PM_2.5_ exposure among outdoor exercisers in Nanjing metropolitan area and identified the factors that are associated with those behaviors. In brief, the level of risk reduction behavior was significantly associated with gender, age, knowledge of PM_2.5_, and whether or not household with children.

### 4.1. Self-Reported the Extent of Knowledge and Risk Reduction Behavior of PM_2.5_

Our study found that although respondents had a good understanding of the risks relating to PM_2.5_, their knowledge of the component of PM_2.5_ was limited. This may be attributed to the government’s lack of publicity on this issue. It is easier for people to access the real-time concentration of atmospheric PM_2.5_ via media. The health hazard of PM_2.5_ is mainly caused by chemicals on particulates [39,40]. However, the data on the composition characteristics of local PM_2.5_ and the impact of mass concentration were rarely mentioned. Understanding the characteristics of PM_2.5_ compositions may encourage local residents to take targeted protective measures of preventing risks from PM_2.5_ exposure. Therefore, it is important for the government and healthcare providers to strengthen educative publicity in this area.

We found avoiding opening windows was the most common risk reduction behavior. This could be explained by people’s risk perceptions of air pollution. People will take actions when they are aware of the risks to their health in the fog and haze from a variety of media sources [41]. Avoiding opening windows seems to be one of the easiest actions to take. However, pollutant particles can still transport indoors via leaks in the building envelope even closing the doors and windows. It was reported that indoor-outdoor concentration ratios are often over 50% [42,43]. Therefore, it may not be one of the most efficient ways to reduce PM_2.5_ exposure. The current studies showed that air purifiers could result in significant reduction in indoor PM_2.5_ concentration and improve human health in some areas with ambient particulate air pollution [44,45]. Two randomized, double-blind cross over trials among Chinese college students suggested clear cardiopulmonary benefits of indoor air purification [44,45]. However, using air purifiers was found to be the least common risk reduction behavior in this study. This could be because air purifiers are unaffordable and their protection effects are doubted by many people. Therefore, the public should be informed of the protective role of air purifiers and be guided to choose a cost-effective air purifier.

In China, AQI has been updated daily to the general public through different medias, including television, radio, newspapers, broadcast telephone messages, and the Internet, as well as the health advice about how to reduce PM_2.5_ exposure [19]. Therefore, it is not surprising that adjusting their physical exercise styles/amounts according to AQI was the second common risk reduction behavior. This is similar to a systematic review by D’Antoni et al., which found that the 9.7% to 57.0% (Mean = 31.0%) of their participants would reduce or reschedule outdoor activities during periods of poor air quality [38]. Wearing a mask was the main risk reduction behavior of PM_2.5_ in many previous studies [25,26]. In this study, wearing a mask was not found to the most common risk reduction behavior but the proportion of wearing them was higher than a previous Chinese study in 2012, which reported that 3.9% of urban residents wore face masks when they perceived bad air quality [23,24]. However, most of our respondents would use medical one-off masks or cotton or gauze masks, which only provide minimal protection against haze [23,26]. Hence, the low proportion of using professional antismog face masks needs to be rectified and guidance on choosing proper face masks should be more available to the public.

### 4.2. Predictor of the High-Level Risk Reduction Behavior

In this study, females were more likely to adopt risk reduction behaviors than males. This finding is somewhat consistent with previous studies. A study comparing Chinese residents’ perceived risks on PM_2.5_ and preventive actions during hazy days found that women were more willing to reduce their amount of outdoor activities and more willing to wear a mask than males [25]. Age is another important factor that influences PM_2.5_ risk reduction behavior. Since the elderly are more sensitive to air pollution and pay more attention to their health and safety than the youth, they were found to be more likely to adopt risk reduction behaviors. Nonetheless, contrasting findings has been reported in another Chinese study that reported participants aged 18–44 were more likely to wear face masks than other older age groups [25]. No significance between age and behavior changes was also found in the study of De Pretto et al., which is related to atmospheric haze pollution [46]. The disagreement about the results of age-related research reminds us that, it is likely that predictors may be affected by sample population and the area surveyed when the risk reduction behavior is evaluated.

In addition, knowledge of PM_2.5_ was found to be a strong predictor of having higher-level risk reduction behavior in this study. This can be partly explained by the COM-B framework which suggested an integrated ‘behavior system’ (B) involving capability (C), opportunity (O), and motivation (M) [37,38]. In this study, we found that people who had higher levels of knowledge were more willing to adapt themselves to low haze exposure, which was similar to a study conducted in Malaysia [46]. Other research conducted in China also found that those with a greater understanding of the health effects of air pollution preferred to wear a mask on hazy days [25].

Households with children were found to be another potential predictor for high-level risk reduction behavior of PM_2.5_. According to the COM-B framework, ‘reflective motivation’ includes the beliefs that air pollution can have negative health impact, and it is also regarded as perceived severity [37,38]. Similarly, a systematic review conducted by D’Antoni et al. showed that when the general public fail to perceive air pollution as a ‘personal’ risk, it is less likely that they will change their behaviors [38]. A meta-analysis conducted by Sheeran et al. also confirmed that when people perceived severity or susceptibility to the threat (if no preventative action is taken), they were more likely to successfully change their behaviors in the behavioral change interventions [47]. In this study, it could be inferred that individuals with a child are more likely to perceive severity and susceptibility to the PM_2.5_ threat and thereby they may take more protect measures in daily life.

### 4.3. Limitations and Strength

This study is the first study to describe multiple risk reduction behavior of PM_2.5_ among outdoor exercisers in a Chinese setting. It provides valuable information to encourage local health-related authorities to take into account the importance of reducing PM_2.5_ exposure on a daily basis at the individual-level and to set up appropriate intervention strategies.

Several limitations to the current study should be acknowledged. Firstly, the study was conducted on convenience sample. Therefore, selection bias and underrepresentation were inevitable. Secondly, responses about the risk reduction behavior and knowledge towards PM_2.5_ were self-reported, which is, by definition, subjective. Thirdly, given that respondents recalled some of their behavior (e.g., some particular behavior on the last haze day) instead of common behavioral habits to adverting PM_2.5_, the potential for recall bias was possible. Hence, objective measurements on knowledge extent regarding PM_2.5_ will be applied in future work. Moreover, predictors of each specific risk reduction behavior will be further investigated to expand the present findings.

## 5. Conclusions

The present study described the multiple risk reduction behaviors regarding PM_2.5_ exposure among outdoor exercisers in the Nanjing metropolitan area. It also reports on how people’s demographic characteristics and knowledge of PM_2.5_ affect their risk reduction behavior. Educational interventions to promote PM_2.5_ protective behavior should target males, aged younger than 50 or even over 71 years old, and households with no children. Our findings also draw attention to the importance of increasing public knowledge of PM_2.5_, as a potential strategy in reducing PM_2.5_ exposure. In brief, this study can be used for policy-makers and healthcare providers to ascertain a clear understanding of individual self-protection strategies to minimize exposure to air pollution, as well as their influencing factors, to improve community awareness in haze polluted areas.

## Figures and Tables

**Table 1:** Socio-demographic characteristics of the study population.

Characteristic	No. (%)	(N = 302)
**Gender**		
Male	52.3	158
Female	47.7	144
**Age group**		
≤50 years old	25.2	76
51–60 years old	27.8	84
61–70 years old	24.5	74
≥71 years old	22.5	68
**Occupation**		
Government staff	35.1	106
Company employee	42.4	128
Self-Employed	22.5	68
**Educational level**		
Junior high school or below	26.5	80
High school	31.1	94
Junior college	19.9	60
College or above	22.5	68
**Whether or not have chronic illness**		
Yes	53.0	160
No	47.0	142
	**No. (%)**	**(N = 300)**
**Whether or not household with chronic illness patients**		
Yes	49.3	148
No	50.7	152
**Living area**		
Urban area	92.7	278
Rural area	2.0	6
Urban-rural marginal area	5.3	16
	**No. (%)**	**(N = 298)**
**Individual income per month**		
≤RMB 3000	27.5	82
RMB 3001–5000	38.9	116
RMB 5001–8000	24.8	74
≥RMB 8001	8.7	26
**Smoking status**		
None-smoker	64.4	192
Ex-smoker	12.8	38
≤10 cigarettes smoked per day	13.4	40
>10 cigarettes smoked per day	9.4	28
**Whether or not household with children**		
Yes	14.8	44
No	85.2	254

**Table 2:** Outdoor physical exercise patterns of the study population.

Characteristic	No. (%)	N
**Average days spent on outdoor physical exercise per week**		298
<1 day	3.4	10
2–3 days	14.1	42
4–5 days	20.8	62
6–7 days	61.7	184
**Average time spent on outdoor physical exercise each time**		298
<30 min	4.7	14
30 min–1 h	36.9	110
1–2 h	43.6	130
>2 h	14.8	44
**Period of outdoor physical exercise**		302
Before 8 a.m.	43.4	131
8 a.m.–1 p.m.	5.6	17
1–6 p.m.	4.6	14
After 6 p.m.	46.4	140
**Physical exercise styles**		302
Walking	39.1	118
Running or brisk walking	23.8	72
Dancing	7.9	24
Two or more ways	29.2	88

**Table 3:** Outcome of multivariate logistic regression analysis (mean value as cutting point) for factors that influence the adoption of risk reduction behavior regarding PM_2.5_ (N = 292).

Characteristic	Lower-Level	Higher-Level	*p*	OR	95% CI	
	No. (%) (N)	No. (%) (N)			Lower	Upper
**Gender**						
Male (Reference)	53.3 (82)	46.8 (72)				
Female	27.5 (38)	72.5 (100)	0.002 **	3.22	1.53	6.78
**Age group**						
≤50 years old (Reference)	50.0 (38)	50.0 (38)				
50–60 years old	23.7 (18)	76.3 (58)	0.030 **	2.53	1.10	5.85
60–70 years old	35.1 (26)	64.9 (48)	0.165	1.78	0.79	4.02
≥71 years old	57.6 (38)	42.4 (28)	0.408	0.70	0.30	1.62
**Occupation**						
Government staff (Reference)	36.0 (36)	64.0 (64)				
Company employee	36.5 (46)	63.5 (80)	0.476	1.35	0.59	3.06
Self-Employed	57.6 (38)	42.5 (28)	0.201	1.65	0.77	3.54
**Educational level**						
Junior high school (Reference) or below	48.7 (38)	51.3 (40)	0.217	0.55	0.21	1.43
High school	40.0 (36)	60.0 (54)	0.809	0.90	0.38	2.13
Junior college	37.9 (22)	62.1 (36)	0.888	0.94	0.37	2.38
College or above	36.4 (24)	63.6 (42)	0.217	0.55	0.21	1.43
**Smoking status**						
None-smoker (Reference)	31.1 (58)	68.9 (128)				
Ex-smoker	50.0 (20)	50.0 (20)	0.288	2.36	0.49	11.43
≤10 cigarettes smoked per day	57.9 (22)	42.1 (16)	0.170	3.51	0.59	21.05
>10 cigarettes smoked per day	71.4 (20)	28.6 (8)	0.719	1.38	0.24	7.93
**Individual income per month**						
≤RMB 3000 (Reference)	35.5 (27)	64.5 (50)	0.945	0.94	0.16	5.52
RMB 3001–5000	42.6 (49)	57.4 (66)	0.453	0.55	0.12	2.62
RMB 5001–8000	49.3 (36)	50.7 (37)	0.473	0.57	0.12	2.66
≥RMB 8001	29.6 (8)	70.4 (19)	0.945	0.94	0.16	5.52
**Whether or not have chronic illness**						
No (Reference)	43.4 (66)	56.6 (86)				
Yes	38.6 (54)	61.4 (86)	0.922	1.04	0.43	2.52
**Whether or not household with chronic illness patients**						
No (Reference)	41.5 (61)	58.8 (86)				
Yes	40.7 (59)	59.3 (86)	0.758	1.11	0.59	2.09
**Whether or not household with children**						
No (Reference)	43.6 (108)	56.5 (140)				
Yes	27.3 (12)	72.7 (32)	0.035 **	2.59	1.07	6.26
**Self-reported the extent of knowledge about PM_2.5_**						
Very low (Reference)	56.3 (36)	43.8 (28)				
Low	46.7 (70)	53.3 (80)	0.116	1.80	0.87	3.76
High	20.0 (12)	80.0 (48)	0.000 **	7.55	2.64	21.60
Very high	11.1 (2)	88.9 (16)	0.005 **	12.70	2.14	75.17

Note: ** Difference is significant at the *p* < 0.05 level (2-tailed).
